# Supplementary material for: The Dental Aesthetic Index and Its Association with Dental Caries, Dental Plaque and Socio-Demographic Variables in Schoolchildren Aged 12 and 15 Years
Source: Int J Environ Res Public Health. 2021 Sep 16;18(18):9741. doi: 10.3390/ijerph18189741 (PMC8468568; doi:10.3390/ijerph18189741)
Supplement: Supplementary file 1 [file ijerph-18-09741-s001.zip › ijerph-1330889-supplementary.pdf]

Supplementary Material

The Dental Aesthetic Index and its Association with Dental Caries, Dental Plaque and Socio-Demographic Variables in Schoolchildren Aged 12 and 15 Years

Paula Fernández-Riveiro, Nerea Obregón-Rodríguez, María Piñeiro-Lamas, Almudena Rodríguez-Fernández, Ernesto Smyth-Chamosa and María Mercedes Suárez-Cunqueiro

Table S1. Differences between age groups: Chi-square test *p*-values.

Variables	Null	Moderate	Severe/Very Severe
Sex	0.572	0.111	0.009
Social class	<0.001	0.001	0.007
Residence	0.186	0.001	0.055
Type of school	0.003	<0.001	<0.001
Caries experience	<0.001	<0.001	0.302
Dental plaque	<0.001	<0.001	<0.001

Table S2. Intraclass Correlation Coefficient index (ICC) between each of the five work teams and the external calibrator.

Work Teams	ICC (IC95%)	*p*-Value
**Work Team 1**		
Number of missing upper teeth	1 (1–1) [a]	-
Number of missing lower teeth	1 (1–1) [a]	-
Crowding (incisal segments)	1 (1–1) [a]	-
Spacing in the incisal segments	1 (1–1) [a]	-
Midline diastema	0.97 (0.95–0.98)	<0.001
Largest anterior maxillary irregularity	0.99 (0.98–0.99)	<0.001
Largest anterior mandibular irregularity	1 (1–1) [a]	-
Anterior maxillary overjet	1 (1–1) [a]	-
Anterior mandibular overjet	1 (1–1) [a]	-
Vertical anterior open bite	1 (1–1) [a]	-
Anteroposterior molar relationship	1 (1–1) [a]	-
Dental Aesthetic Index (DAI) Score	0.99 (0.98–1)	<0.001
**Work Team 2**		
Number of missing upper teeth	1 (1–1) [a]	-
Number of missing lower teeth	1 (1–1) [a]	-
Crowding (incisal segments)	0.90 (0.83–0.95)	<0.001
Spacing in the incisal segments	1 (1–1) [a]	-
Midline diastema	1 (1–1) [a]	-
Largest anterior maxillary irregularity	0.99 (0.98–0.99)	<0.001
Largest anterior mandibular irregularity	0.98 (0.94–0.98)	<0.001
Anterior maxillary overjet	0.99 (0.99–1)	<0.001
Anterior mandibular overjet	1 (1–1) [a]	-
Vertical anterior open bite	1 (1–1) [a]	-
Anteroposterior molar relationship	1 (1–1) [a]	-

DAI Score	0.99 (0.99–1)	<0.001
**Work Team 3**		
Number of missing upper teeth	1 (1–1) [a]	-
Number of missing lower teeth	0.80 (0.64–0.88)	<0.001
Crowding (incisal segments)	1 (1–1) [a]	-
Spacing in the incisal segments	0.88 (0.79–0.93)	<0.001
Midline diastema	0.96 (0.94–0.98)	<0.001
Largest anterior maxillary irregularity	0.97 (0.95–0.98)	<0.001
Largest anterior mandibular irregularity	0.98 (0.94–0.98)	<0.001
Anterior maxillary overjet	0.98 (0.97–0.99)	<0.001
Anterior mandibular overjet	1 (1–1) [a]	-
Vertical anterior open bite	1 (1–1) [a]	-
Anteroposterior molar relationship	0.93 (0.88–0.96)	<0.001
DAI Score	0.94 (0.90–0.97)	<0.001
**Work Team 4**		
Number of missing upper teeth	1 (1–1) [a]	-
Number of missing lower teeth	1 (1–1) [a]	-
Crowding (incisal segments)	1 (1–1) [a]	-
Spacing in the incisal segments	1 (1–1) [a]	-
Midline diastema	1 (1–1) [a]	-
Largest anterior maxillary irregularity	0.99 (0.98–0.99)	<0.001
Largest anterior mandibular irregularity	0.97 (0.94–0.98)	<0.001
Anterior maxillary overjet	1 (1–1) [a]	-
Anterior mandibular overjet	1 (1–1) [a]	-
Vertical anterior open bite	1 (1–1) [a]	-
Anteroposterior molar relationship	0.93 (0.88–0.96)	<0.001
DAI Score	0.99 (0.98–0.99)	<0.001
**Work Team 5**		
Number of missing upper teeth	1 (1–1) [a]	-
Number of missing lower teeth	1 (1–1) [a]	-
Crowding (incisal segments)	1 (1–1) [a]	-
Spacing in the incisal segments	1 (1–1) [a]	-
Midline diastema	0.96 (0.94–0.98)	<0.001
Largest anterior maxillary irregularity	0.98 (0.96–0.99)	<0.001
Largest anterior mandibular irregularity	0.95 (0.91–0.97)	<0.001
Anterior maxillary overjet	1 (1–1) [a]	-
Anterior mandibular overjet	1 (1–1) [a]	-
Vertical anterior open bite	1 (1–1) [a]	-
Anteroposterior molar relationship	0.88 (0.78–0.93)	<0.001
DAI Score	0.98 (0.96–0.99)	<0.001

[a] 100% agreement.

Table S3. Cohen's Kappa concordance index between each of the five work teams and the external calibrator.

Work Teams	Weighted Kappa	
	K_W (95% CI)	*p*-Value
**Work Team 1**		
Malocclusion: four categories [a]	1 (1–1) [b]	-
Malocclusion: yes/no	1 (1–1) [b]	-
**Work Team 2**		
Malocclusion: four categories [a]	1 (1–1) [b]	-
Malocclusion: yes/no	1 (1–1) [b]	-
**Work Team 3**		
Malocclusion: four categories [a]	0.84 (0.81–0.87)	<0.001
Malocclusion: yes/no	0.78 (0.74–0.82)	<0.001
**Work Team 4**		
Malocclusion: four categories [a]	1 (1–1) [b]	-
Malocclusion: yes/no	1 (1–1) [b]	-
**Work Team 5**		
Malocclusion: four categories [a]	0.94 (0.93–0.96)	<0.001
Malocclusion: yes/no	0.92 (0.90–0.95)	<0.001

[a] No malocclusion, moderate, severe or very severe malocclusion.
[b] 100% agreement.

Table S4. Intraclass Correlation Coefficient index (ICC) between each of the five work teams.

Work Teams	ICC (95% CI)	*p*-Value
**Work Team 1**		
Number of missing upper teeth	1 (1–1) [a]	-
Number of missing lower teeth	1 (1–1) [a]	-
Crowding (incisal segments)	0.96 (0.93–0.98)	<0.001
Spacing in the incisal segments	0.93 (0.88–0.96)	<0.001
Midline diastema	0.95 (0.91–0.97)	<0.001
Largest anterior maxillary irregularity	0.98 (0.96–0.99)	<0.001
Largest anterior mandibular irregularity	0.80 (0.64–0.89)	<0.001
Anterior maxillary overjet	0.93 (0.88–0.96)	<0.001
Anterior mandibular overjet	0.93 (0.88–0.96)	<0.001
Vertical anterior open bite	1 (1–1) [a]	-
Anteroposterior molar relationship	0.81 (0.66–0.89)	<0.001
DAI Score	0.93 (0.88–0.96)	<0.001
**Work Team 2**		
Number of missing upper teeth	1 (1–1) [a]	-
Number of missing lower teeth	1 (1–1) [a]	-
Crowding (incisal segments)	0.84 (0.71–0.91)	<0.001
Spacing in the incisal segments	0.88 (0.79–0.93)	<0.001
Midline diastema	0.91 (0.85–0.95)	<0.001
Largest anterior maxillary irregularity	0.97 (0.94–0.98)	<0.001
Largest anterior mandibular irregularity	0.96 (0.93–0.98)	<0.001
Anterior maxillary overjet	0.93 (0.87–0.96)	<0.001
Anterior mandibular overjet	1 (1–1)[a]	-

Vertical anterior open bite	1 (1–1) [a]	-
Anteroposterior molar relationship	0.84 (0.72–0.91)	<0.001
DAI Score	0.93 (0.88–0.96)	<0.001
**Work Team 3**		
Number of missing upper teeth	1 (1–1) [a]	-
Number of missing lower teeth	1 (1–1) [a]	-
Crowding (incisal segments)	0.79 (0.62–0.88)	<0.001
Spacing in the incisal segments	0.96 (0.92–0.98)	<0.001
Midline diastema	0.54 (0.18–0.74)	0.014
Largest anterior maxillary irregularity	0.93 (0.87–0.96)	<0.001
Largest anterior mandibular irregularity	0.95 (0.90–0.97)	<0.001
Anterior maxillary overjet	0.84 (0.72–0.91)	<0.001
Anterior mandibular overjet	1 (1–1) [a]	-
Vertical anterior open bite	1 (1–1) [a]	-
Anteroposterior molar relationship	0.75 (0.56–0.86)	<0.001
DAI Score	0.93 (0.88–0.96)	<0.001
**Work Team 4**		
Number of missing upper teeth	1 (1–1) [a]	-
Number of missing lower teeth	1 (1–1) [a]	-
Crowding (incisal segments)	0.80 (0.64–0.89)	<0.001
Spacing in the incisal segments	0.94 (0.89–0.97)	<0.001
Midline diastema	0.75 (0.58–0.84)	<0.001
Largest anterior maxillary irregularity	0.94 (0.89–0.96)	<0.001
Largest anterior mandibular irregularity	0.96 (0. 93–0.98)	<0.001
Anterior maxillary overjet	0.89 (0.81–0.94)	<0.001
Anterior mandibular overjet	1 (1–1) [a]	-
Vertical anterior open bite	1 (1–1) [a]	-
Anteroposterior molar relationship	0.88 (0.80–0.94)	<0.001
DAI Score	0.91 (0.85–0.95)	<0.001
**Work Team 5**		
Number of missing upper teeth	1 (1–1) [a]	-
Number of missing lower teeth	1 (1–1) [a]	-
Crowding (incisal segments)	0.94 (0.90–0.97)	<0.001
Spacing in the incisal segments	0.80 (0.64–0.88)	<0.001
Midline diastema	0.76 (0.60–0.85)	<0.001
Largest anterior maxillary irregularity	0.96 (0. 93–0.98)	<0.001
Largest anterior mandibular irregularity	0.95 (0. 90–0.97)	<0.001
Anterior maxillary overjet	0.94 (0. 89–0.96)	<0.001
Anterior mandibular overjet	1 (1–1) [a]	-
Vertical anterior open bite	0.88 (0. 79–0.93)	<0.001
Anteroposterior molar relationship	0.94 (0.90–0.97)	<0.001
DAI Score	0.96 (0.92–0.98)	<0.001

[a] 100% agreement.

Table S5. Cohen's Kappa concordance index between each of the five work teams.

**Work Teams**	**Weighted Kappa**	
	**K_W (95% CI)**	***p*-Value**
**Work Team 1**		
Malocclusion: four categories [a]	0.84 (0.81–0.87)	<0.001
Malocclusion: yes/no	0.82 (0. 78–0.86)	<0.001
**Work Team 2**		
Malocclusion: four categories [a]	0.84 (0.80–0.89)	<0.001
Malocclusion: yes/no	0.72 (0.66–0.78)	<0.001
**Work Team 3**		
Malocclusion: four categories [a]	0.73 (0.67–0.79)	<0.001
Malocclusion: yes/no	0.79 (0.72–0.85)	<0.001
**Work Team 4**		
Malocclusion: four categories [a]	0.65 (0.55–0.76)	<0.001
Malocclusion: yes/no	0.65 (0.55–0.76)	<0.001
**Work Team 5**		
Malocclusion: four categories [a]	0.85	<0.001
Malocclusion: yes/no	1 (1–1) [a]	-

[a] No malocclusion, moderate, severe, or very severe malocclusion.
